# The Early Postoperative Complications of Two Different Tube Ligation Methods in Baerveldt Implant Surgery

**DOI:** 10.5005/jp-journals-10008-1170

**Published:** 2015-01-15

**Authors:** Shuri Kawamorita, Teruhiko Hamanaka, Testurou Sakurai

**Affiliations:** Consultant, Department of Ophthalmology, Japanese Red Cross Medical Center, Japan; Consultant, Department of Ophthalmology, Japanese Red Cross Medical Center, Japan; Instructor, Department of Center of General Education, Tokyo University of Science, Suwa, Japan

**Keywords:** Glaucoma, Baerveldt, Tube ligation method, Absorbable ligation method, Nonabsorbable ligation method, Tube shunt surgery.

## Abstract

**Objective:** To investigate the early postoperative complications in two different tube ligation methods during the first 3 months in Baerveldt implant surgery.

**Participants:** This study involved 157 eyes from 144 patients who underwent Baerveldt Implant Surgery at the Japanese Red Cross Medical Center, Tokyo, Japan.

**Methods:** Pre- and postoperative intraocular pressure (IOP), combined surgery, postoperative time-point of tube ligation release, and postoperative complications in two different tube ligation methods [absorbable ligation method using 8-0 polyglactin suture (group A) and nonabsorbable ligation method using 7-0 nylon suture (group B)] were retrospectively reviewed.

**Results:** After excluding eyes that had undergone combined trabeculectomy (26 eyes) and vitrectomy (2 eyes), eyes with previous tube surgery (22 eyes), and eyes that had undergone the stent method (1 eye), 30 of 28 patients in group A and 71 eyes of 71 patients in group B were found to fit the criteria of this study. The rate of successful surgical outcome was 80% in group A and 74.6% in group B (p = 0.705). During the 3 months postoperative, high IOP tended to occur more often in group B (67.6%) than in group A (46.7%) (p = 0.073), and ciliochoroidal detachment tended to occur more often in group A (10.0%) than group B (2.8%) (p = 0.154).

**Conclusion:** The results of this study show that both ligation methods are effective, however, the selection of tube ligation method should be done in accordance with the different method-specific risks to which may occur.

**How to cite this article:** Kawamorita S, Hamanaka T, Sakurai T. The Early Postoperative Complications of Two Different Tube Ligation Methods in Baerveldt Implant Surgery. J Curr Glaucoma Pract 2014;8(3):96-100.

## INTRODUCTION

For the treatment of glaucoma, two types of implants for tube-shunt surgery are currently available; a valve-type implant that directs the aqueous humor to the sub-Tenon’s capsule immediately after the operation, and valveless-type implant that directs the aqueous humor to the sub-Tenon’s capsule after waiting for approximately 1 month postoperative for the capsule formation around the plate. To avoid excessive postoperative filtration, the valveless-type implant requires ligation of the tube at the time of surgery.

In regard to tube ligation, there are currently three available methods: the (1) absorbable ligation method,^1,3^ the (2) nonabsorbable ligation method,^2,4^ and the (3) stent method.^3^ Serious complications associated with tube shunt surgery usually occur within the first postoperative month.^12^ Thus, the purpose of this present study was to retrospectively investigate and compare the complications and surgical outcomes of the absorbable ligation method and the nonabsorbable ligation method for the treatment of glaucoma during the first 3 months after surgery, i.e. the early postoperative period.

## METHODS

### Study Design and Patients

The records of 144 consecutive patients (157 total eyes) who underwent Baerveldt® BG 250 or 350 Glaucoma Implant (Abbot Medical Optics, Abbot Park, IL) surgery combined with mitomycin C instillation at the Japanese Red Cross Medical Center, Tokyo, Japan, between February 2008 and December 2012, were retrospectively reviewed. Informed consent was obtained from all patients in this study, and all surgeries were performed by a single surgeon (TH). The eyes were divided into the following two tube ligation methods: (1) absorbable ligation using 8-0 polyglactin suture (Group A) and (2) nonabsorbable ligation using 7-0 nylon suture (Group B). In the patients who underwent Baerveldt surgery from February 2008 to October 2011, the tube was ligated with nonabsorbable suture (Group B) and in the other patients who underwent that surgery from November 2011 to December 2012, the tube was ligated with absorbable suture (Group A). We retrospectively investigated the data of the two groups [i.e. combined operations, preoperative intraocular pressure (IOP), postoperative IOP at 1 day, 1 week, 1 month and 3 months postoperative and at the time of the tube release as well as any postoperative complications]. In all patients, the following items were examined at the time of the follow-up: anterior segment of the eye by use of a slit-lamp, fundus, IOP, and the presence or absence of the most peripheral ciliochoroidal detachment (CCD) by use of anterior segment optical coherence tomography (AS-OCT). Patient who had undergone Baerbeldt implant surgery via the stent method, a previous tube implant surgery, combined surgery with trabeculectomy, pars plana Baerveldt, or in which a postoperative complication of plate or tube exposure occurred were excluded from the study. This study was approved by the Institutional Review Board of the Japanese Red Cross Medical Center.

### Surgical Technique

After making a fornix-based conjunctival incision at the inferior or superior position, the Tenon’s capsule and sclera were exposed to identify two adjacent rectus muscles. After determining the area where the plate would be installed, 0.04% Mitomycin C was applied to that area for 3 minutes and then washed away. The plate was then installed under the two adjacent rectus muscles and fixed with 8-0 nylon suture. In group A, the tube was completely ligated at the distance of 5 mm before the plate using 8-0 polyglactin suture. The tube was fixed at 3 places on the sclera using 8-0 nylon suture, and 3 Sherwood slits were then created using the 8-0 polyglactin needle. The penetration of the tube was made with width of 1.5 to 2 times of the needle. The tube was then inserted into the anterior chamber from either the 6 or 12-o’clock position after making an introduction hole for the tube at the distance of 1.5 mm away from the limbus using a 23-guage needle. In group B, the tip of the tube was ligated using 7-0 nylon suture, inserted into the anterior chamber from either the 6 or 12-o’clock position, and then fixed at 3 places on the sclera using 8-0 nylon suture.^5^ The tube was then covered with preserved sclera and the conjunctiva was then sutured in both methods.

Success or failure of the surgical outcome was defined as follows: at least 20% reduction of IOP and an IOP ≥ 6 mm Hg and ≤ 21 mm Hg was defined as a definite success; at least 20% reduction of IOP or an IOP ≥ 6 and ≤ 21 mm Hg was defined as a qualified success, and an IOP ≤ 5 and ≥ 25 mm Hg was defined as a failure. The tube release time in group A was defined as the point of time when the IOP dropped more than 6 mm Hg from the previous IOP.

**Table 1:** **Table 1:** Excluded eyes

Combined surgery eyes: trabeculectomy		26 eyes	
Pars plana Baerveldt		2 eyes	
Eyes having already undergone a		22 eyes	
tube-shunt surgeries			
Stent method eyes		1 eye	
Complication of tube or plate exposure		6 eyes	
Total		57 eyes	

**Table 2:** **Table 2:** Demographic data and clinical characteristics

				*Group A* *(n = 30)*		*Group B* *(n = 71)*		*Total* *(n = 101)*		*p-value*	
Age (mean)				13-93 (61.7)		18-93 (62.58)				0.825	
Gender		Male		24 (80)		51 (71.8)		75		0.744	
n (%)		Female		6 (20)		20 (28.2)		26		0.628	
Glaucoma diagnosis		Primary open angle		13 (43.3)		26 (36.6)		39		0.687	
n (%)		Pseudo-exfoliation		6 (20)		13 (18.3)		19		1.000	
		Neovascular		2 (6.7)		12 (16.9)		14		0.343	
		Uveitic		3 (10)		7 (9.9)		10		1.000	
		Posner-Schlossman syndrome		0 (0)		3 (4.2)		3		0.555	
		Traumatic		0 (0)		3 (4.2)		3		0.555	
		Chronic angle closure		0 (0)		2 (2.8)		2		1.000	
		Steroid		1 (3.3)		3 (4.2)		4		1.000	
		Congenital		2 (6.7)		0 (0)		2		0.094	
		Pigmentary		1 (3.3)		0 (0)		1		0.304	
		After keratoplasty		2 (6.7)		1 (1.4)		3		0.223	
		After cataract operation		0 (0)		1 (1.4)		1		1.000	
High myopia n (%)				5 (16.7)		10 (14.1)		15		0.769	
Preoperative		≤ 21 mm Hg		4 (13.3)		10 (14.1)		14		0.751	
IOP		> 21 mm Hg		25 (80)		61 (85.9)		86		0.872	
n (%)		Mean ± SD		27.27 ± 6.84		30.66 ± 10.04		29.65 ± 9.30		0.053	
Plate size		250		19 (63.3)		20 (28.2)		39		0.048	
n (%)		350		11 (36.7)		51 (71.8)		62		0.097	
Number of glaucoma		Preoperative		2.50 ± 0.63		2.68 ± 0.81				0.243	
medications (mean ± SD)		Postoperative		1.20 ± 1.21		1.73 ± 1.34				0.058	

High myopia: -6.00 diopters and more

### Statistical Analysis

All statistical analyses were performed using R software version 2.13.2. We used the two-sided Student t-test for continuous variables and Fisher exact test for categorical variables. For the tube release time, the Kolmogorov-Smirnov test was used. A p-value of < 0.05 was considered statistically significant.

**Table 3:** **Table 3:** Postoperative data

		*Group A (%)* *n = 30*		*Group B (%)* *n = 71*		*Total (%)* *n = 101*		*p-value*	
*Success/failure*								0.705	
Definite		24 (80)		53 (74.6)		77 (76.2)			
Qualified		4 (13.3)		9 (12.7)		12 (12.9)			
Failure		2 (6.7)		9 (12.7)		11 (10.9)			
*HYPO during 3M*								1.000	
+		7 (23.3)		16 (22.5)		23 (22.8)			
-		23 (76.7)		55 (77.5)		78 (77.2)			
*HYPO at 3M*								1.000	
+		0 (0)		1 (1.4)		1 (1)			
-		30 (100)		70 (98.6)		100 (99)			
*OHT during 3M*								0.073	
+		14 (46.7)		48 (67.6)		62 (61.4)			
-		16 (53.3)		23 (32.4)		39 (38.6)			
*OHT at 3M*								0.719	
+		2 (6.7)		8 (11.3)		10 (9.9)			
-		28 (93.3)		63 (88.7)		91 (90.1)			
*CCD at 3M*								0.154	
+		3 (10)		2 (2.8)		5 (5)			
-		27 (90)		69 (97.2)		96 (95)			
*Complications*								0.705	
+		1 (3.3)		2 (2.8)		3 (3)			
-		29 (96.7)		69 (97.2)		98 (97)			

Definite: More than 20% reduction of IOP and less than 21 mm Hg; Qualified: More than 20% reduction of IOP or less than 21 mm Hg; Failure: Less than 6 mm Hg or more than 25 mm Hg; HYPO: Ocular hypotension; OHT: Ocular hypertension; CCD: Ciliochoroidal detachment

**Table 4:** **Table 4:** Surgical outcomes in relation to the difference of plate size

*Groups*		*250 (%)*		*350 (%)*		*Total*		*p-value*	
*Group A*									
D		14 (74)		10 (91)		24		0.647	
Q		3 (16)		1 (9)		4			
F		2 (11)		0 (0)		2			
*Group B*									
D		11 (55)		42 (82)		53		0.054	
Q		4 (20)		5 (10)		9			
F		5 (25)		4 (8)		9			
*Group A + B*									
D		25 (64)		52 (84)		77		0.075	
Q		7 (18)		6 (10)		13			
F		8 (18)		4 (6)		11			

D: Definite; Q: Qualified; F: Failure

## RESULTS

A total of 156 eyes of 143 patients underwent Baerveldt^®^ BG implant surgery (250 or 350) at the Japanese Red Cross Medical Center between February 2008 and December 2012. The absorbable ligation method, nonabsorbable ligation method, stent method, and pars plana Baerveldt were applied in 40 eyes, 114 eyes, 1 eye, and 1 eye respectively. Eyes excluded from the study included 26 eyes of 25 patients that had undergone a combined surgery with trabeculectomy, 2 eyes with pars plana Baerveldt, 1 eye that underwent the stent method, and 22 eyes of 16 patients with a previous history of glaucoma tube surgery, and six cases of six eyes in which postoperative tube exposure occurred (Table 1). Ultimately, a total of 101 eyes (30 eyes of 28 patients in group A and 71 eyes of 71 patients in group B) were investigated in this study. Demographic data and clinical characteristics are summarized in Table 2.

No statistically significant differences in success rate were found between the two groups; i.e. 24 eyes (80%) in group A and 53 eyes (74.6%) in group B (p = 0.705, Table 3). Moreover, no statistically significant differences related to plate size were found between the two groups (p = 0.075, Table 4). At 3 months postoperative, ocular hypotension occurred in 1 eye in group B. No statistically significant differences in ocular hypertension were found between the two groups during the 3 months postoperative, however, it tended to occur less often in group A (46.7%) than in group B (67.6%) (p = 0.073). In addition, no statistically significant differences in the rate of CCD (as observed by AS-OCT) was found between the two groups at 3 months postoperative, yet it tended to be lower in group B (2.8%) than in group A (10.0%) (p = 0.154). Except for 1 eye in group A, CCD was observed only in the extremely periphery in all cases. Kissing choroidal detachment was observed in the posterior pole in 1 eye in group A. Other postoperative complications included shallow anterior chamber in 1 eye and tube occlusion in one eye in group B. As for the eye with shallow anterior chamber, plate exposure occurred after massaging the eye due to uncontrolled IOP after releasing the tube ligation. As for the tube occlusion, the inflammatory products developed on the tip of the tube at 7 weeks postoperative. As ocular hypertension occurred at the 11th postoperative week, removal of the tissue that developed on the tip of the tube was subsequently performed.

**Graph 1:**
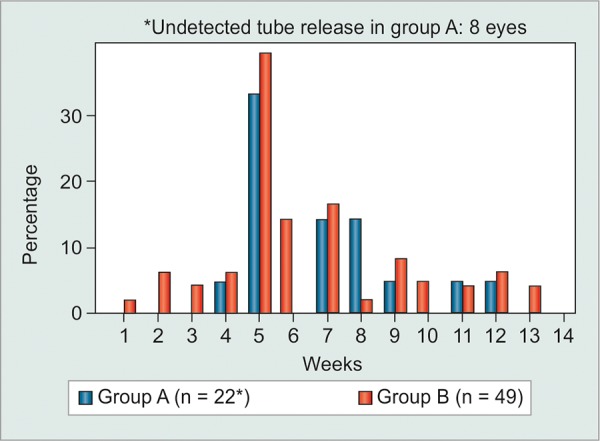
The time of tube release during the 3-month postoperative period following Baerveldt implant surgery

**Table 5:** **Table 5:** Undetected tube release and persistence of tube obstruction

*Glaucoma type*		*Eye*	
Undetected tube release in group A		8	
Secondary		3	
Primary open-angle		2	
Neovascular		1	
Pigmentary		1	
Capsular		1	
Persistence of tube obstruction in group B		22	
Primary open-angle		10	
Capsular		4	
Neovascular		2	
Uveitis		2	
Posner-Schlossman syndrome		2	
Chronic angle closure		1	
Traumatic		1	
High myopia		4	

In both groups, the tube was mostly released in approximately the 5th postoperative week. The mean postoperative time of tube ligation release was 6.9 weeks in group A and 6.3 weeks in group B (Graph 1) (p = 0.6044). Since, tube release was performed with a laser in the nonabsorbable ligation cases, the IOP did not decrease immediately after releasing and some cases needed a few days until the IOP decreased. In eight eyes in group A, the time of the tube ligation release was not detected due to not being able to observe the greater than 5 mm Hg drop in IOP at the successive follow-up periods. Tube ligation was not released in 22 eyes of group B during the observational period because the postoperative IOP remained good (Table 5).

## DISCUSSION

In the absorbable ligation method, the tube is ligated with 8-0 polyglactin suture and the tube is expected to release naturally within 4 to 6 weeks after the surgery. This method has the advantage of escaping ocular hypertension due to the Sherwood slit^6^ being made, however, the time of tube release cannot be controlled or adjusted. On the other hand, in the nonabsorbable ligation method, the tube is ligated with nonabsorbable suture and the tube is manually released by laser suture lysis (LSL) at the appropriate time. However, the nonabsorbable ligation method has some disadvantages, as the ligature knots make it difficult to insert the tube into the anterior chamber^5^ and the Sherwood slit effect is not available for this method.

The rates of ocular hypotension during the first 3 months postoperative did not differ in both groups, yet CCD tended to occur more often in group A (group A: 10.0%, group B: 2.8%, p = 0.154, Table 3). Kissing choroidal detachment was observed in 1 eye in group A. We theorized that the tube ligation probably released around the 5th week postoperative in that eye, but the patient continued taking antiglaucoma medications even after tube ligation release. The CCD was refractory and still existed in the 3rd postoperative month.

No statistically significant differences in ocular hypertension were found between the two groups during the 3 months postoperative period, however, it tended to occur more often in group B than in group A (group A: 46.7%, group B: 67.6%, p = 0.073, Table 3). The higher incidence of ocular hypertension observed in group B may be due to the fact that the Sherwood slit effect is not available when using the nonabsorbable ligation method.

Plate size may have an influence on the effectiveness of tube surgery.^7^ In our study, different plate sizes of BG 250 and 350 were unevenly selected for use in group A and, in group B, which may have affected the IOP lowering effect in both groups. However, recent studies have shown no significant difference between BG 250 and 350 with regard to complications which affect the results and the long-term outcome.^8,14^

In 22 cases (30.0%) in group B, the tubes were not released, even at 3 months postoperative, due to the IOP being well-maintained. In cases of uveitis^9,10^ and high myopia,^11^ a natural release of the tube ligature is unfavorable, as it is more possible for ocular hypotension and choroidal detachment to occur after the tube releases. In addition, if the patient’s conjunctiva is thin or is incised near the plate, leakage of aqueous humor may occur or the plate may become exposed through the incised conjunctiva after the tube ligation release. Therefore, it may be better to select the nonabsorbable ligation method in such cases. When LSL is used to manually release of the tube, it is sometimes experienced that the tube is not completely released just after the procedure. This may be because the Baerveldt tube is soft and has poor shape-memory, unlike that of a Molteno tube. In addition, the Tenon’s capsule must be lifted by aqueous humor in order to form a bleb over the Baerveldt plate. This is possibly the reason why the IOP does not become lower immediately after the tube is released.

The findings of the tube *vs* trabeculectomy (TVT) study reported by Gedde et al showed that after 5 years of follow-up, the most prevalent postoperative complications were choroidal effusion (16%), persistent corneal edema (16%), and shallow or flat anterior chamber (11%).^12^ In the Ahmed Baerveldt comparison study reported by Budenz et al, the numerous postoperative complications included a shallow or flat anterior chamber (23%), hyphema (18%), and tube obstruction (14%).^13^ In this present study, we found serious choroidal detachment (0.99%) in group A, and a shallow anterior chamber (0.99%) (due to suture separation of the conjunctiva after eye massage) and tube obstruction (0.99%) in group B. The tube obstruction observed in the 1 eye in group B was due to the proliferative tissue, which may be caused by an inflammatory reaction resulting from tube-to-iris contact.

In summary, the findings of this study showed no significant differences in the postoperative outcome between eyes that underwent the absorbable ligation method and those that underwent the nonabsorbable ligation method. However, CCD tended to occur at a higher rate in the absorbable ligation method and ocular hypertension tended to occur at a higher rate in the nonabsorbable ligation method. Thus, these results indicate that the absorbable ligation method should be avoided in eyes with uveitis and high myopia which have a higher risk of postoperative ocular hypotension, in eyes with thin conjunctiva, and in eyes which require a conjunctival suture near the plate. On the other hand, the nonabsorbable ligation method should be avoided in eyes with advanced glaucoma with severely deteriorated visual function, because the Sherwood slit effect is not available with that method. It may be important to select the tube ligation method according to the above-mentioned eye-specific circumstances, yet further study is needed in regard to complications, associated with the two different ligation methods in the long-term postoperative period.
